# Comparative Metabolomics Analysis Reveals the Unique Nutritional Characteristics of Breed and Feed on Muscles in Chinese Taihe Black-Bone Silky Fowl

**DOI:** 10.3390/metabo12100914

**Published:** 2022-09-27

**Authors:** Xinjun Liao, Xiaowen Shi, Hongmei Hu, Xiangju Han, Kai Jiang, Yong Liu, Guanghua Xiong

**Affiliations:** 1 College of Life Sciences, Jinggangshan University, Ji’an 343009, China; 2 Ji’an Key Laboratory of Genetics, Breeding and Reproduction in Taihe Silky Fowl, Ji’an Innovation Center of Science and Technology Development, Ji’an 343099, China; 3 Key Laboratory of Embryo Development and Reproductive Regulation of Anhui Province, Fuyang Normal University, Fuyang 236041, China

**Keywords:** Taihe silky fowl, metabolic components, un-targeted metabolome, breed and feed, biosynthesis of amino acids

## Abstract

The Chinese Taihe Black-bone silky fowl (TBsf) is the homology of medicine and food and has high nutritional and medical value all over the world. However, the nutritional compositions and potential metabolite biomarkers of Taihe silky fowl in muscles are still poorly understood. In this study, we investigated the differences in nutritional components between TBsf and another similar breed (Black Feathered chicken and laid green-shelled eggs, BF-gsc). Meanwhile, we also explored the divergences in muscle characteristics of Taihe silky fowl fed with two different diets; that is, normal chicken feed (TBsf-ncf) and *Broussonetia papyrifera*-fermented feed (TBsf-bpf). Firstly, the growth performance and biochemical index of Taihe silky fowl was significantly different compared with black-feathered chicken. Secondly, we identified the metabolic alterations in Taihe silky fowl by performing an un-targeted UHPLC-Q-TOF-MS/MS analysis. Our results suggested that all the metabonomic characteristics had obvious separation between TBsf-ncf, TBsf-bpf and BF-gsc groups, both in the positive and negative ion mode by PCA analysis. Next, OPLS-DA multivariate analysis revealed that 57 metabolites (in positive mode) and 49 metabolites (in negative mode) were identified as differential metabolites between the TBsf-ncf and BF-gsc groups. These differential metabolites were mainly enriched to ABC transporters, biosynthesis of amino acids and aminoacyl-tRNA biosynthesis. Besides, 47 metabolites (in positive) and 13 metabolites (in negative) were differentially regulated between the TBsf-ncf and TBsf-bpf groups, which were majorly involved in histidine metabolism and linoleic metabolism. Furthermore, the integrated network analysis suggested that DL-arginine, DL-isoleucine, linoleoylcarnitine, stearoylcarnitine (positive) and ricionleic acid, D-proline, and uric acid (negative) were the significant metabolic biomarkers in Taihe silky fowl. Moreover, the metabolites of primaquine, ticlpoidine, riboflavin, acetylcarnitine (positive) and salicylic acid, acetaminophen sulfate, and glutamic acid (negative) were markedly changed in the Taihe silky fowl fed with BP-fermented feed. In summary, a global survey of the nutritional components and metabolite differences was performed in muscle tissues of Taihe silky fowl between various breeds and feeds. Meanwhile, our study provided valuable information for nutritional components and metabolic biomarkers in Chinese Taihe silky fowl, which greatly promoted the economic value of the black-boned chicken industry and laid a solid theoretical foundation for the development of chicken products with greater added value in future.

## 1. Introduction

Black-bone silky fowl (*Gallus gallus domesticus Brisson*), is a chicken originating from Taihe County, east of Wushan Mountain in the Jiangxi Province of China, where it has been raised for more than 2000 years [1,2]. The distinct features of Taihe black-bone silky fowl (TBsf) are the snow-white feathers and the presence of melanin in various organs such as skin, meat and bones, when compared with the other common chickens [3,4]. TBsf has a highly nutritive value and pharmaceutical action, which is known as the marvel of traditional Chinese medicine for various ailments. According to previous reports, TBsf has certain medicinal value to cure headache, hepatitis, asthma and other heart diseases [5,6]. At the molecular level, TBsf contains the higher levels of carnosine in the mixed meat and breast meat than other chickens [7,8]. Besides, natural melanin is considered as one of the most important components in TBsf, which has a wide range of biochemical activities such as anti-oxidation, free radical-scavenging and immunomodulatory effects [9,10]. However, the overall metabolite profile of Chinese Taihe black-bone silky fowl and its metabolic differences compared with other chickens has not been fully investigated up to now.

In order to fully utilize the economic value of black-bone silky fowl, many previous studies have conducted explorations from the perspective of genetics and molecular biology [11]. For example, whole genome resequencing revealed that the EDN3 gene might interact with the ncRNA to generate melanin in Xichuan black-bone chicken [12]. The Chinese indigenous chickens had great phenotypic variation and possessed more genetic diversity than that reported in many other countries [13,14]. In addition, environmental factors such as nutrition and stress may also have an important role in modifying muscle traits [15,16]. For example, feeding chickens with the olive oil-supplemented diet could increase the expression of avian uncoupling protein in chicken muscle [17]. Meanwhile, *Broussonetia papyrifera* (BP) belongs to the family of Moraceae, which is normally used as high-quality feed ingredients for livestock animals due to the high nutritional value, content of crude protein, lysine and methionine [18,19]. The addition of BP-fermented feed could significantly improve growth performance and meat quality in sheep [20]. BP-fermented feed can significantly increase the WPS-2 and actinobacteria, and result in changes in the intestinal microbes of laying hens [21]. From all the above studies, it is suggested that different breeds and feeds may lead to differences in muscle nutrients of chickens, but the change characteristics of these metabolites affected by breed and feed in Taihe black-bone silky fowl have been largely unknown.

Metabolomics is the comprehensive study describing the whole of the endogenous metabolites in various organisms or tissues, and is widely used in a variety of fields including biomedicine and food science to identify novel metabolic biomarkers [22,23]. Metabolome and other “omics” such as proteomics, transcriptomics and genomics frequently represent the global assessment of metabolite levels in a biological sample [24,25]. Metabolome can be divided into non-targeted and targeted metabolome, and non-targeted metabolome is generally based on liquid chromatography (LC) and mass spectrometry (MS) technology to conduct qualitative and quantitative analysis of metabolites in the samples at the same time [26,27]. Non-targeted metabolomics can reflect the overall metabolite levels and have been widely used to reveal the dynamic characteristics of metabolites under different conditions [28,29]. At present, the UHPLC-Q-TOF-MS/MS technique has been widely used to determine the levels of metabolites in animals [30,31]. Several previous studies have been reported concerning the metabolite changes in chicken eggs by using un-targeted LC-MS analysis [32,33]. However, the muscle metabolites of Taihe silky fowl influenced by the breed and feed traits have not been explored at the metabolome level.

Taihe black-bone silky fowl has many unique properties compared with other chickens. In this study, we systematically analyzed the metabolite differences in muscle tissues from three different breed and feed conditions (TBsf-ncf, TBsf-bpf and BF-gsc). We mainly used the UHPLC-Q-TOF-MS/MS technique to identify the differentially metabolites responsible for its unique phenotype in Taihe black-bone silky fowl. In addition, many metabolites were identified and related enriched signaling pathways were determined in the Taihe silky fowl. For the first time, we demonstrated that both the genetic and environmental factors are critical to determining muscle composition in Taihe silky fowl, which should be considered in the efforts to meet consumer needs and develop nutritionally functional chickens in future.

## 2. Materials and methods

### 2.1. Feeding and Determination of the Growth Performance in Laboratory Animals

In this experiment, the chickens of TBsf-ncf and TBsf-bpf were obtained from Wangbeitu Taihe silky fowl Development Co., Ltd. and Aoxin Taihe silky fowl Development Co., Ltd., respectively. Meanwhile, the chickens of BF-gsc were acquired from the local farm of Ji’an city. They were introduced into the laboratory animal room of Jinggangshan University at 8 weeks of age. After introduction, all chickens were reared in individual cages (78 cm L × 50 cm W × 60 cm H) under a photoperiod cycle of 16 h L/8 h D, which freed access to diet and water, respectively. Besides, at least 20 chickens of each breed were fed in our lab and used for this experiment. The chickens of TBsf-ncf and BF-gsc were fed with normal chicken feed, while the chickens of TBsf-bpf were fed with BP-fermented feed. On the other hand, the normal chicken feed mainly contains 60% corn, 20% soybean meal and 10% wheat bran. The BP-fermented feed was prepared as follows: approximately 0.5 g of *Enterococcus faecalis* was added into the 1 kg *B. papyrifera* powder and the mixture was fermented at 30 °C for 20 days. The chickens of TBsf-bpf were fed with 5% BP-fermented feed replacing the corn and soybean meal in the normal diet. Furthermore, the chicken breeding and experimental procedures were strictly carried out following the guidelines approved by the Independent Animal Care Committee (IACC) of Jinggangshan University (JGSU-IACC20210028).

Approximately 12 weeks after the chickens were introduced into the lab, the indexes of growth performation of these chickens, including body weight and feed intake, were detected and calculated in each group. Meanwhile, the laying time of these chickens was at the 20th week (about 140 days after birth) and 20 eggs from each kind of chicken were used for egg weight calculations. Besides, the chickens produced eggs in the special egg-laying boxes (25 cm L × 25 cm W × 30 cm H) set in each cage, with some dry and soft leaves together with wheat straw placed in the nest. During the experimental period, eggs were collected at 17:00 every day, and the weight of eggs in each group was recorded in detail. Furthermore, the biochemical parameters such as the contents of triglyceride were measured using the detection kit (CAS No. F001-1-1, Institute of Nanjing Jiancheng Bioengineering, Nanjing, China) according to the previous protocols in our lab [34,35].

### 2.2. Collection and Preparation of Chicken Samples

To investigate the effects of breed and feed on metabolome, we compared the metabolites differences in two breeds (Taihe silky fowl and Black feather chicken) and two feeds (Normal chicken feed and BP-fermented feed). In order to identify the nutritional components in different breeds of chicken, we killed four chickens of each breed (TBsf-ncf, TBsf-bpf and BF-gsc), and three muscle tissues from each chicken were randomly selected for subsequent experiments (that is, 12 samples including four biological replicates × three technical replicates in each group). After that, these samples were frozen by liquid nitrogen and kept at −80 °C until needed for further analysis.

Next, the chicken samples were slowly thawed on ice. In addition, about 50 mg of muscle tissue was homogenated with three volumes of cold methanol/acetonitrile/H_2_O (2:2:1) to precipitate proteins. The mixtures were adequately vortexed and subjected to ultrasound for 30 min, then incubated at −20 °C for 10 min. After that, the samples were centrifuged and re-dissolved in 100 μL of acetonitrile/water (1:1, *v/v*) for mass spectrometry loading analysis. On the other hand, the pooled QC sample was also used for control analysis. We employed QC samples to evaluate the system stability and performance before sample loading and during the whole experimental process.

### 2.3. UHPLC-Q-TOF-MS/MS Analysis

The metabolites of chicken samples in each group were separated by using UHPLC system (Agilent 1290 Infinity LC) with HILIC column, which consisted of solvent A (water + 25 mM ammonium acetate + 25 mM ammonia) and solvent B (acetonitrile). The gradient elution began at 95% B from 0–0.5 min, and B linearly decreased to 65% from 0.5–7 min, linearly reduced to 40% from 7–8 min, and maintained at 40% from 8–9 min. Next, B linearly rose from 40% to 95% at 9–9.1 min, and maintained at 95% from 9.1–12 min. During the whole process, the samples were placed at the 4℃ automatic sampler. Meanwhile, the QC samples were used to evaluate the reliability of experimental data.

The mass spectrometry conditions of Q-TOF were as follows: the samples were analyzed by the AB Triple TOF 6600 system (AB SCIEX, Framingham, MA, USA), and positive and negative ion mode were detected by the electrospray ionization (ESI). The setting parameters of ESI source were the following: auxiliary heating gas 1 (gas1), 60 psi; auxiliary heating gas 2 (gas2), 60 psi; Collision energy (CE) was set at 35 ± 15 eV, and the declustering potential (DP) was set at ±60 V. The secondary mass spectrum was obtained by information-dependent acquisition (IDA) mode and screened by peak intensity value.

### 2.4. Quality Assessment and Differential Metabolites Identification

The raw data with an instrument-specific format (.wiff) were converted into the standard format (.mzXML) by the ProteoWizard MS converter tool [36]. Next, peak alignment and area extraction were performed by using XCMS online tools [37]. After that, structural identification of metabolites was performed based on the accuracy of the *m/z* value (<10 ppm) and MS/MS data that were matched to our self-built database. Furthermore, MetaboAnalyst 5.0 was employed for further statistical analysis [38].

After normalization, the processed data were used for multivariate analysis such as PCA and OPLS-DA. The evaluation parameters of the model (R^2^Y, Q^2^) were obtained through 7-fold cross-validation, and the permutation test was used to measure the effectiveness of the model. The variable importance in the projection (VIP) value in the OPLS-DA model and Student’s *t*-test were applied to evaluate the significance of differential metabolites. We defined that the metabolites with statistically significant difference were the VIP value > 1 and *p* value < 0.05.

### 2.5. Functional Enrichment and Correlation Analysis of Differential Metabolites

Based on the differential metabolites identified in various comparing conditions, KEGG pathway analysis was conducted to investigate the putative metabolomics pathways affected by the effects of breed and feed in Taihe silky fowl. Hierarchical clustering of differential metabolites in mainly enriched pathways was performed by using heatmap.2 function within gplots package in R environment. Pearson’s correlation coefficient was used to evaluate the association between breed and feed in differential metabolites. A correlation coefficient |r| ≥ 0.8 and *p* ≤ 0.05 was considered to reflect a high correlation. To make a visual representation, the metabolites were selected and constructed the regulatory enrichment network by using Cytoscape software.

### 2.6. Statistical Analysis

SPSS v.20.0 (Chicago, IL, USA) and GraphPad v.7.0 were used for all statistical analysis and figure drawing, respectively. An independent sample T-test or one-way analysis of variance (ANOVA) followed by a Dunnett’s post hoc test were used to evaluate the mean differences between Taihe silky fowl and black feather chickens. All the experiments contained at least three biological repeats and the results were expressed as means ± standard deviation (SD). Statistical significance was considered as * *p* < 0.05 and ** *p* < 0.01.

## 3. Results

### 3.1. The Growth Performance and Biochemical Components of Chinese Taihe Silky Fowl

**Figure 1:**
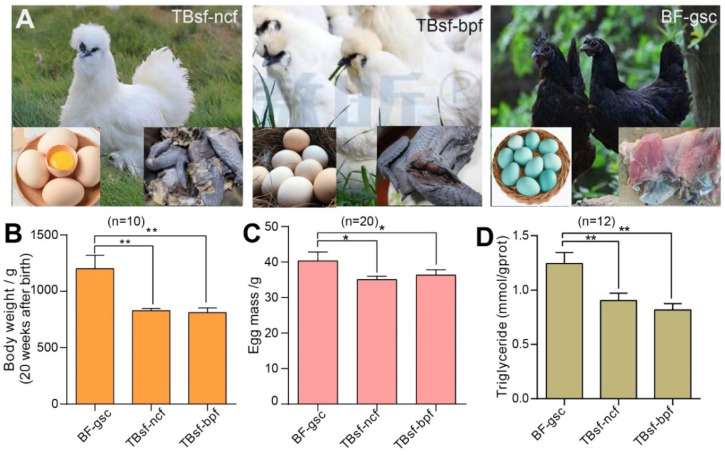
The growth performance and biochemical component of Taihe silky fowl was significantly different from Black-feathered chicken. (**A**) The morphological characteristics of each chicken in TBsf-ncf, TBsf-bpf and BF-gsc, respectively. The egg was shown in the lower left corner and meat color was shown in the lower right corner. (**B**) The body weight of three kinds of chicken at 20 weeks after birth was measured in each group (n = 10). (**C**) The egg mass of three kinds of chicken was calculated in each group (n = 20). (**D**) The contents of triglyceride (TG) in three kinds of chicken were detected in blood samples (n = 12, four biological replicates * three techniques replicates). For all experiments, the value was represented as mean ± S.D. * *p* < 0.05, ** *p* < 0.01. Abbreviations: Taihe black-bone silky fowl fed with normal chicken feed (TBsf-ncf); Taihe black-bone silky fowl fed with *Broussonetia papyrifera*-fermented feed (TBsf-bpf); Black-feathered chicken and laid green-shelled eggs (BF-gsc).

To investigate the effects of breed and feed on meat quality in Chinese Taihe silky fowl, we used three kinds of chickens: Taihe Black-bone silky fowl fed with normal chicken feed (TBsf-ncf); Taihe Black-bone silky fowl fed with *Broussonetia papyrifera*-fermented feed (TBsf-bpf); and Black-feathered chicken with green-shelled eggs (BF-gsc). The morphological characteristics, egg diversity and muscle color in each breed of chicken are shown in Figure 1A. Firstly, we evaluated the growth state of each chicken and our results suggested that the body weight at 20 weeks after birth in Taihe silky fowl (TBsf-ncf, 823.4 ± 66.7 g; TBsf-bpf, 851.2 ± 82.8 g) was significantly lower than that of black-feathered chicken (BF-gsc, 1156.1 ± 106.3 g) (Figure 1B). Meanwhile, the egg mass of Taihe silky fowl also reduced compared with black-feathered chicken (39.6 ± 5.7 g), but there was no significant difference between TBsf-ncf (36.4 ± 3.8 g) and TBsf-bpf (37.1 ± 4.1 g) (Figure 1C). Besides, other growth indicators such as feed intake, egg production and feed conversion ratio were also calculated in the Supplemental Table S3.

Triglyceride (TG) is an important component of blood lipids, which is mainly synthesized by adipose tissue in the liver and small intestine. The content of TG in chickens is an important indicator to determine whether this food is suitable for special people including diabetics and hyperlipidemia. Therefore, we evaluated the difference in serum biochemical indexes of these chickens, and our results revealed that the contents of TG in Taihe silky fowl were also sharply decreased from those of black-feathered chicken (Figure 1D). Taken together, these results demonstrated that the growth index of Taihe silky fowl were significantly different from black-feathered chicken and Taihe silky fowl may be more used for food by patients with diabetics and hyperlipidemia.

### 3.2. Multivariate Statistical Analysis of the Untargeted Metabolomics Data

**Figure 2:**
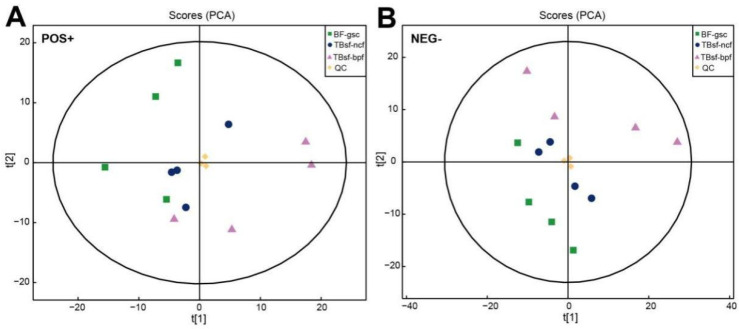
Quality assessment of UHPLC-Q-TOF-MS/MS metabolomic data in Taihe silky fowl. (**A**) The PCA scores of the chicken muscle samples in each group with the ESI positive ion mode. (**B**) The PCA scores of the chicken muscle samples in each group with the ESI negative ion mode. T[1] represents principal component 1 and T[2] represents principal component 2, which the aggregation degree of QC samples reflects the repeatability of the experimental data. Abbreviations: green dots indicate the BF-gsc samples, blue dots indicate the TBsf-ncf samples, purple dots indicate the TBsf-bpf samples and yellow dots indicate the QC samples.

For the non-targeted metabolomics analysis, the processed data with removed low-quality values were normalized by the log transformation and Pareto scaling. The molecular features extracted from all experimental samples and quality control (QC) samples were analyzed by the principal component analysis (PCA). Our results showed that three QC samples were tightly clustered in the PCA space for the electrospray ionization (ESI), both in the positive and negative mode, respectively (Figure 2A,B). The consistently repeated QC injections indicated the excellent reliability and stability of the experimental procedure in metabolomics analysis. Moreover, in order to evaluate the global expression levels of muscle metabolites in three kinds of black-bone chicken samples, the unsupervised PCA method was applied to detect the degree of dispersion between the two groups of samples. The results demonstrated that there were significant differences compared with TBsf-ncf, TBsf-bpf and BF-gsc groups in the 2D PCA plots (Supplementary Figure S1). It is worth mentioning that the variation degree of four biological replicates samples in the TBsf-bpf group was greater than that in the TBsf-ncf group, which suggested that *Broussonetia papyrifera*-fermented feed may greatly affect the metabolite difference in muscle tissue in Taihe silky fowl.

**Figure 3:**
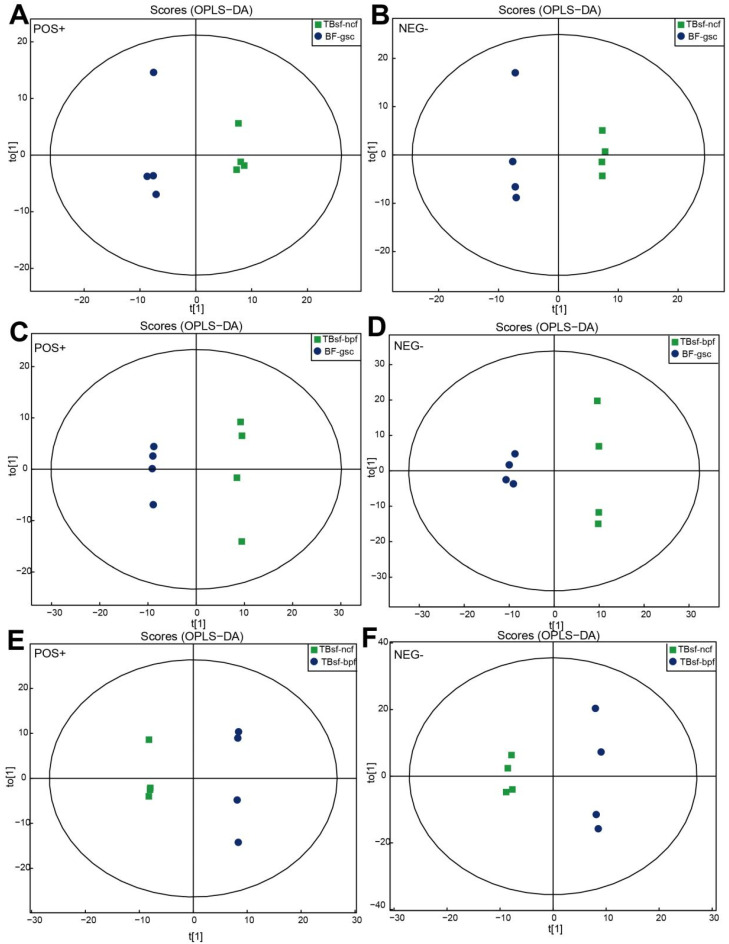
OPLS-DA plots derived from UHPLC-Q-TOF-MS/MS spectra in Taihe silky fowl. (**A**,**B**) The OPLS-DA score map between TBsf-ncf (green dots) and BF-gsc group (blue dots) both in the positive and negative ion mode, respectively. (**C**,**D**) The OPLS-DA score map between TBsf-bpf (green dots) and BF-gsc group (blue dots) both in the positive and negative ion mode, respectively. (**E**,**F**) The OPLS-DA score map between TBsf-ncf (green dots) and TBsf-bpf group (blue dots) both in the positive and negative ion mode, respectively.

Orthogonal partial least-squares discrimination analysis (OPLS-DA) maximizes the difference between groups in T[1], while the orthogonal principal component to [1] reflects the variation within the group, which has good sample classification and reliable predictive ability. As shown in Figure 3A,B, there was a clear separation between the TBsf-ncf and BF-gsc groups based on the OPLS-DA plot, both in the positive ion mode (R^2^Y = 0.995; Q^2^ = 0.735) and negative ion mode (R^2^Y = 0.999, Q^2^ = 0.751). Similarly, there was a significant difference between the TBsf-bpf and BF-gsc groups, both in the positive ion mode (R^2^Y = 0.999; Q^2^ = 0.739) and negative ion mode (R^2^Y = 0.997, Q^2^ = 0.732) (Figure 3C,D). Besides, OPLS-DA score maps between the TBsf-ncf and TBsf-bpf groups were presented both in the positive ion mode (R^2^Y = 0.995; Q^2^ = 0.735) and negative ion mode (R^2^Y = 0.997, Q^2^ = 0.694), which suggested that different feeds affected the metabolite components in Taihe silky fowl (Figure 3E,F). In summary, the above results indicated that there were obvious separation characteristics between Taihe silky fowl and black-feathered chicken, which further demonstrated that the metabolites of Taihe silky fowl exhibited a certain degree of alterations in muscle tissues compared with other chickens.

### 3.3. Identification of Differential Metabolites in Muscle Tissues of Taihe Silky Fowl

**Figure 4:**
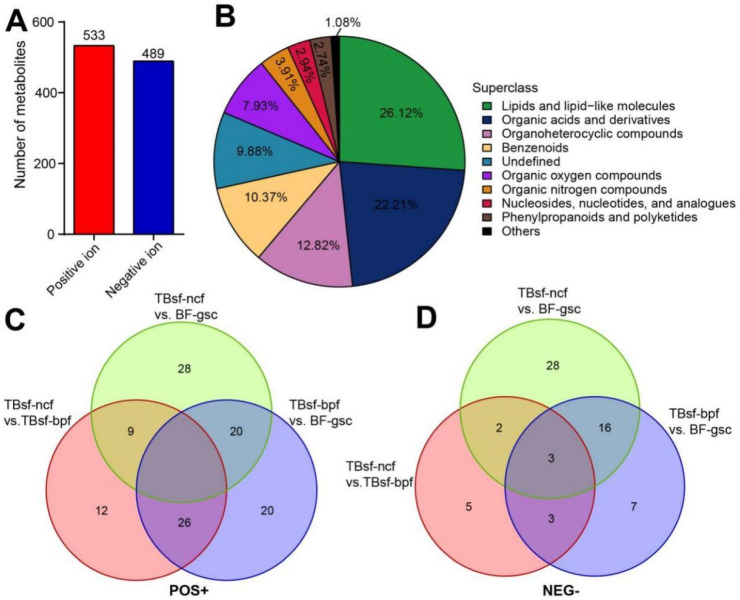
The differential metabolites were identified in Taihe silky fowl by metabolomic analysis. (**A**) The number of metabolites extracted from positive and negative ion peaks in HILIC column were presented. (**B**) The proportion of identified metabolites in each chemical classification. The specific chemical categories of each metabolite can be found in the legend, and the proportion of each superclass was presented in the corresponding pie chart. (**C**) Venn diagram showing the shared and unique differential metabolites between Taihe silky fowl and black-feathered chicken in positive ion mode. (**D**) Venn diagram showing the shared and unique differential metabolites between Taihe silky fowl and black-feathered chicken in negative ion mode. The overlapping regions represent metabolites that are concomitantly regulated in two or three samples.

The metabolites in three kinds of chicken samples were identified by matching with the retention time, molecular weight, secondary fragmentation and other information in our local database. A total of 14,410 molecular features were extracted from HILIC column, in which 1022 metabolites were identified, including 533 in positive ion mode and 489 in negative ion mode (Figure 4A). The detailed results of metabolite identification are shown in Supplemental Table S1. All metabolites identified in this experiment were classified and counted according to their chemical taxonomy, and the proportion of various metabolites was shown in Figure 4B. Our results suggested that the top three main metabolite molecules in chicken muscle samples were lipids and lipid-like molecules (26.12%, 267), organic acids and derivatives (22.21%, 227) and organoheterocyclic compounds (12.82%, 131).

The variables importance for the projection (VIP) obtained from the OPLS-DA model can be used to filter metabolites with little change in each group; this could screen the differential metabolic molecules with biological significance. In this metabolomics analysis, we used the strict screening criteria (OPLS-DA VIP > 1 and *p* value < 0.05) as the metabolites with significant differences. Based on the statistical analysis and the VIP value in the OPLS-DA model, 57 metabolites (in positive mode) and 49 metabolites (in negative mode) were identified as significantly differential biomarkers in the TBsf-ncf compared with the BF-gsc group. For example, linoleoylcarnitine (positive, fold change = 8.98) and coniferyl aldehyde (negative, fold change = 10.45) were identified as the top differential metabolites in TBsf-ncf chickens, which can effectively inhibit our fat accumulation and anti-inflammatory effect. Furthermore, 66 metabolites (positive) and 49 metabolites (negative) compared TBsf-bpf with the BF-gsc group, and 47 metabolites (positive) and 13 metabolites (negative) comparing the TBsf-ncf with the TBsf-bpf group were also identified. For examples, riboflavin (positive) and acetaminophen sulfate (negative) were significantly up-regulated in the TBsf-bpf compared with TBsf-ncf chickens; these are important substances involved in redox reaction and peripheral blood vessel expansion. The detailed names and related information of these differential metabolites between the two groups are described in Supplemental Table S2. Besides, the relative expression levels of differential metabolites were also displayed in the hierarchical clustering analysis, which suggested that the number of the up-regulated metabolites accounted for the majority in Taihe silky fowl but not the black-feathered chicken (Supplemental Figure S2).

Next, the overlapping relationship of different metabolites in three kinds of chicken samples was further analyzed by Venn diagram. These results suggested that the differential metabolites in the positive ion mode had a higher overlapping proportion than those in the negative ion mode (Figure 4C,D). There were 20 metabolites in positive ion mode and 16 metabolites in negative ion mode, shared between TBsf-ncf vs. BF-gsc and TBsf-bpf vs. BF-gsc, respectively. Interestingly, the number of common metabolites shared in all three comparison groups was none in the positive and only three in the negative ion mode, which further indicating that Taihe silky fowl showed greater metabolites differences than black-feathered chicken in muscle tissues.

### 3.4. Functional Enrichment Analysis of Differential Metabolites in Taihe Silky Fowl

**Figure 5:**
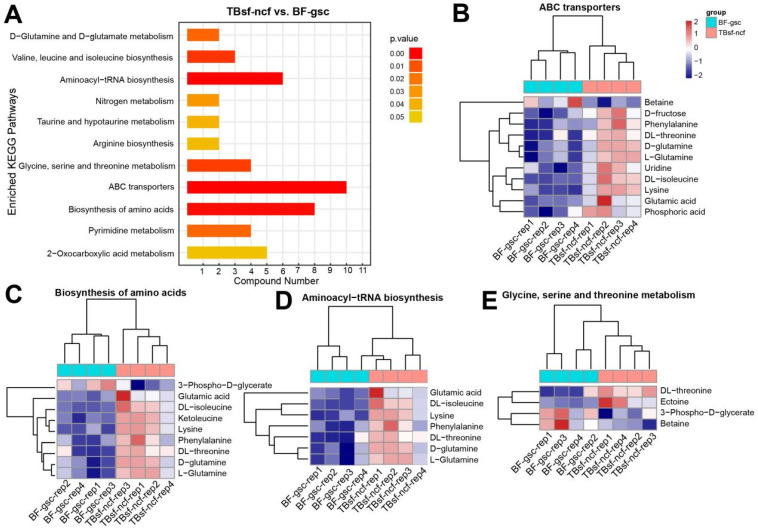
The KEGG enrichment analysis revealed the pathways of protein synthesis and transport were mainly activated in Taihe silky fowl. (**A**) The KEGG pathway enrichment analysis of differential metabolites in Taihe silky fowl compared with the black-feathered chicken. (**B**–**E**) Hierarchical clustering analysis of differential metabolites in ABC transporters, biosynthesis of amino acids, aminoacyl-tRNA biosynthesis, and glycine, serine, and threonine metabolism, respectively.

To further evaluate the molecular function of these differential metabolites in Taihe silky fowl, all the differential components in each group were mapped into the KEGG database. KEGG enrichment analysis suggested that these differential metabolites were mainly enriched in the following metabolic pathways (TBsf-ncf vs. BF-gsc): ABC transporters, biosynthesis of amino acids, aminoacyl-tRNA biosynthesis, valine and leucine and isoleucine biosynthesis, glycine and serine and threonine metabolism, and pyrimidine metabolism (Figure 5A). Bioinformatics analysis revealed that these abnormal metabolites were majorly involved in protein transmembrane transport, translation and amino acid metabolism.

The hierarchical clustering analysis of these representative metabolites in ABC transporters are shown in Figure 5B. These results suggested that most metabolite components such as (D- and L-) glutamine, uridine, glutamic acid, D-fructose, phenylalanine in ABC transporters were significantly up-regulated in TBsf-ncf groups. However, betaine is an amino acid that has potential benefits for helping promote muscle gain and fat loss, and the expression levels of which were significantly decreased in the TBsf-ncf samples. Amino acids are the precursors for the synthesis of many metabolites and our results revealed that most of the metabolites in biosynthesis of amino acids were increased in the TBsf-ncf samples, which suggested that the ability of Taihe silky fowl to synthesize protein and other substances is stronger than the black-feathered chicken (Figure 5C). Similarly, aminoacyl-tRNAs are the substrates for translation and all the components in this pathway were activated in the TBsf-ncf group compared with the BF-gsc group (Figure 5D). Meanwhile, threonine is an essential amino acid, which animals cannot synthesize. Here, we reported that the metabolites of DL-threonine and ectoine in glycine, serine and threonine catabolic pathway were up-regulated in muscle tissues while the metabolites of 3-phospho-D-glycerate were down-regulated in the TBsf-ncf group (Figure 5E). In summary, these results further demonstrated that Taihe silky fowl had significant improvement in protein synthesis and amino acid transport compared with black-feathered chicken.

### 3.5. Integrated Regulatory Networks of Differential Metabolites in Taihe Silky Fowl

**Figure 6:**
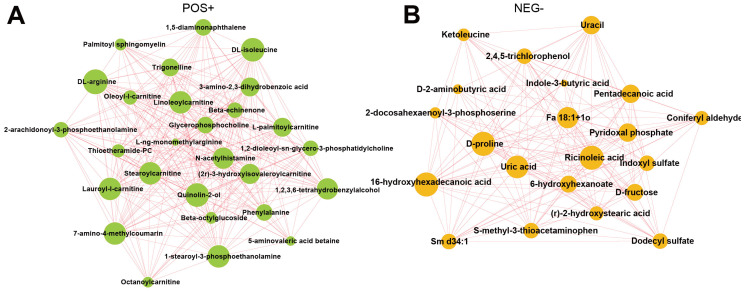
The integrated networks showed the correlation in various differential metabolites of Taihe silky fowl. (**A**) Network data integration and visualization of differential metabolites of Taihe silky fowl in the positive ion mode. (**B**) Network data integration and visualization of differential metabolites of Taihe silky fowl in the negative ion mode. The circle represents the significantly enriched differential metabolites. The size of the circle is related to the degree of connectivity: the larger the degree, the larger the circle.

The interaction between differential metabolites can be shown by integrated regulatory network diagram using the Cytoscape software. Our results suggested that the substrates for protein synthesis, such as DL-arginine and DL-isoleucine, were significantly enriched in the positive mode in the TBsf-ncf samples compared with the BF-gsc group (Figure 6A). Besides, stearoylcarnitine is a fatty ester lipid molecule and acts as a metabolomics biomarker for preeclampsia, and stearoylcarnitine was also obviously enriched in the positive mode. On the other hand, ricionleic acid, proline, uric acid and 6-hydroxyhexadecanoic acid were significantly enriched in the negative ion mode in the Taihe silky fowl (Figure 6B). These results further demonstrated that lipids and organic acids were greatly differential regulated in the muscle tissues of the Taihe silky fowl.

### 3.6. BP-Fermented Feed Induced the Differential Metabolites in Taihe Silky Fowl

**Figure 7:**
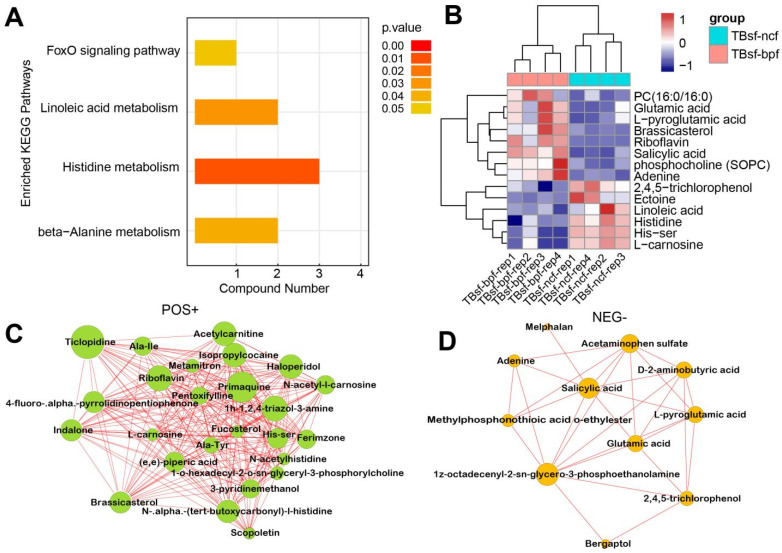
The functional analysis of differential metabolites in Taihe silky fowl fed by BP-fermented feed. (**A**) The KEGG pathway enrichment analysis of differential metabolites in Taihe silky fowl fed with *Broussonetia papyrifera*-fermented feed compared with normal chicken feed. (**B**) The hierarchical clustering analysis of differential metabolites between TBsf-ncf and TBsf-bpf groups. (**C**) The integrated networks of enriched differential metabolites in the positive ion mode between TBsf-ncf and TBsf-bpf groups. (**D**) The integrated networks of enriched differential metabolites in the negative ion mode between TBsf-ncf and TBsf-bpf groups.

Restrictive feeding influences the systemic metabolism of nutrients in some animals, while this effect has not been evaluated in chickens. Therefore, we investigated the effect of *BP-fermented* feed (TBsf-bpf group) compared with normal chicken feed (TBsf-ncf group) in Taihe silky fowl. KEGG enrichment analysis suggested that histidine metabolism, linoleic acid metabolism and beta-alanine metabolism were significantly enriched in the TBsf-bpf sample compared with the TBsf-ncf sample (Figure 7A). Moreover, the cluster analysis revealed that linoleic acid, histidine, 2,4,5-trichlorophenol were significantly down-regulated while glutamic acid, L-pyroglutamic acid and brassicasterol were significantly up-regulated in the TBsf-bpf group (Figure 7B). Besides, functional network analysis suggested that primaquine, riboflavin, acetylcamitine and ticlpoidine were significantly enriched in the positive ion mode (Figure 7C). Meanwhile, salicylic acid, acetaminophen sulfate, and 3-phosphoethanolamine were significantly enriched in the negative ion mode (Figure 7D). From these results, it is suggested that *Broussonetia papyrifera-fermented* feed can significantly change the metabolite composition in Taihe silky fowl.

## 4. Discussion

Taihe silky fowl is a National Protection of Geographical Indications that received numerous international prizes, and which has many nutritional and medicinal benefits [39,40]. This study aimed to investigate the effects of breed and feed on two kinds of Taihe silky fowl and one kind of black-feathered chicken. Our results have shown that there are significant differences such as body weight, egg mass and eggshell color between Taihe silky fowl and black-feathered chicken. Meanwhile, the contents of triglyceride in Taihe silky fowl (TBsf-ncf and TBsf-bpf) were also significantly lower than those in black-feathered chicken. Interestingly, there was no significant difference in growth and development characteristics in Taihe black chicken fed with normal feed and BP-fermented feed, respectively. Previous studies have suggested that BP-fermented feed had no significant impact on improved egg production and egg quality [21]. In addition, other studies have shown that *Broussonetia papyrifera* fermented feed could decrease the mRNA expression levels of pro-inflammatory cytokines and affect the intestinal antioxidant capacity and microbiota in grass carp [41]. Therefore, whether genetic and environmental factors including different feed conditions can change the muscle quality of Taihe black chicken is a topical issue worthy of our study.

With the development of high-throughput sequencing technologies, high-resolution mass spectrometry plays an important role in metabolite detection due to its higher resolution and sensitivity [42]. Additionally, the rapid UHPLC-Q-TOF-MS/MS metabolome technique was successfully employed to elaborate multiple metabolites and metabolic profile in Taihe silky fowl. The PCA method can reflect the overall distribution of metabolic profiles and variability between and within the sample groups [43]. Our results suggested that despite the four biological repeats in each group being relatively discrete, they are basically clustered in one region in the PCA plots. OPLS-DA is a supervised statistical method that establishes the relationship model between metabolite expression and classification of samples, which can remove the irrelevant variations contained within the mass spectrum and improve effectiveness of the model [44,45]. Thus, OPLS-DA was conducted to confirm the separation and identify the potential biomarkers between Taihe silky fowl and black-feathered chicken. Our results suggested that the metabolites were significantly regulated by both genetic and environmental factors.

Metabolomics analysis identified 57 differential metabolites in the positive mode and 49 differential metabolites in the negative mode between TBsf-ncf and BF-gsc groups. These differential metabolites were majorly related to protein anabolism and fatty acid oxidation. The KEGG pathway analysis revealed that the ABC transporters, biosynthesis of amino acids, and aminoacyl-tRNA biosynthesis were significantly enriched in the Taihe silky fowl compared with black-feathered chicken. Meanwhile, most substrates in protein synthesis such as glutamic acid, threonine and glutamine were significantly up-regulated in the TBsf-ncf group. Betaine is a nutrient contained in many foods, and can prevent fatty liver, cancer and hypertension; however, the expression levels of which was clearly decreased in the Taihe silky fowl. On the other hand, 47 metabolites (positive) and 13 metabolites (negative) were identified as differentially expressed in the TBsf-bpf and TBsf-ncf groups. Functional analysis suggested that histidine metabolism and linoleic acid metabolism were significantly enriched in TBsf-bpf compared with the TBsf-ncf group. Linoleic acid is a long-chain polyunsaturated fatty acid (PUFA) and an essential nutrient in a wide range of animals including chickens, which plays an important role in immunity and reproduction [46,47]. Linoleic acid is one kind of essential fatty acids, which is necessary for the normal growth and development of humans and can only be obtained from food [48,49]. In addition, linolenic acid can be converted into DHA and EPA in vivo, which are extremely beneficial to humans and could reduce the risk of cardiovascular and cerebrovascular diseases [50,51]. Our results revealed that the levels of linoleic acid in BP-fermented feed chickens were significantly lower than those in Taihe silky fowl fed by normal diet; this suggested that BP-fermented feed could significantly improve the nutritional composition of Taihe silky fowl in muscle tissues. Altogether, these results further demonstrated that different genetics and diets may alter muscle metabolite status in Taihe silky fowl.

The metabolic proxies between significantly different metabolites and the regulatory relationships between metabolites in the biological process can be evaluated by correlation analysis [52,53]. In this study we found that many amino acids were significantly enriched in the positive mode, and some lipid molecules were significantly enriched in the negative mode in the Taihe silky fowl. For example, ticlopidine is a potent inhibitor of platelet aggregation induced by ADP and acetylcarnitine is a natural substance that exists in the body, especially in muscle and brain [54,55]. Our results revealed that BP-fermented feed could significantly induce the change in ticlopidine and acetylcamitine in the positive mode in the Taihe silky fowl. Meanwhile, salicylic acid is an endogenous growth regulator of phenolic nature, which participates in the regulation of physiological processes in animals [56]. Our results suggested that salicylic acid and acetaminophen were also significantly enriched in the negative mode of BP-fermented feed chickens. In the modern chicken industry, hybrid chickens (rather than pure breeds) are often used for meat production [57,58]. This study indicates that the combination of breed and feed should be considered to modulate metabolite levels in muscles. Taken together, we found that the effects of breed and feed play an important role in body performance and metabolite components in the Taihe silky fowl. In the meantime, we firstly demonstrated that both genetic and environmental factors were critical to determining muscle composition, which should be considered to meet consumer needs and develop nutritional and functional chickens.

## 5. Conclusions

In this study, we explored the breed and feed effects on the nutritional components in Chinese Taihe silky fowl. Our studies suggested that Taihe silky fowl has significant differences in protein synthesis and amino acid transport compared with black-feathered chicken. In addition, different diets may also significantly change the muscle composition in Taihe silky fowl. In summary, these metabolomics data provide the basic information of muscle metabolites in Taihe black-bone silky fowl, which is helpful to improve the potential economic value of Taihe silky fowl in the future.

## Data Availability

The data presented in this study are available in Supplementary Materials.

## Supplementary Materials

The following supporting information can be downloaded at: https://www.mdpi.com/article/10.3390/metabo12100914/s1, Figure S1: The PCA score map of UHPLC-Q-TOF-MS/MS spectra compared with the two groups both in the positive ion mode and negative ion mode; Figure S2: The cluster analysis of differential metabolites in Taihe silky fowl both in the positive and negative ion modes; Table S1: The metabolites were identified in chicken samples from UHPLC-Q-TOF-MS data both in the positive and negative ion mode; Table S2: List of differential metabolites between the two groups by UHPLC-Q-TOF MS analysis; Table S3: The growth performation of each breed chickens used in this study.
