# Supplementary figures and images for: Comparative Metabolomics Analysis Reveals the Unique Nutritional Characteristics of Breed and Feed on Muscles in Chinese Taihe Black-Bone Silky Fowl
Source: Metabolites. 2022 Sep 27;12(10):914. doi: 10.3390/metabo12100914 (PMC9611261; doi:10.3390/metabo12100914)

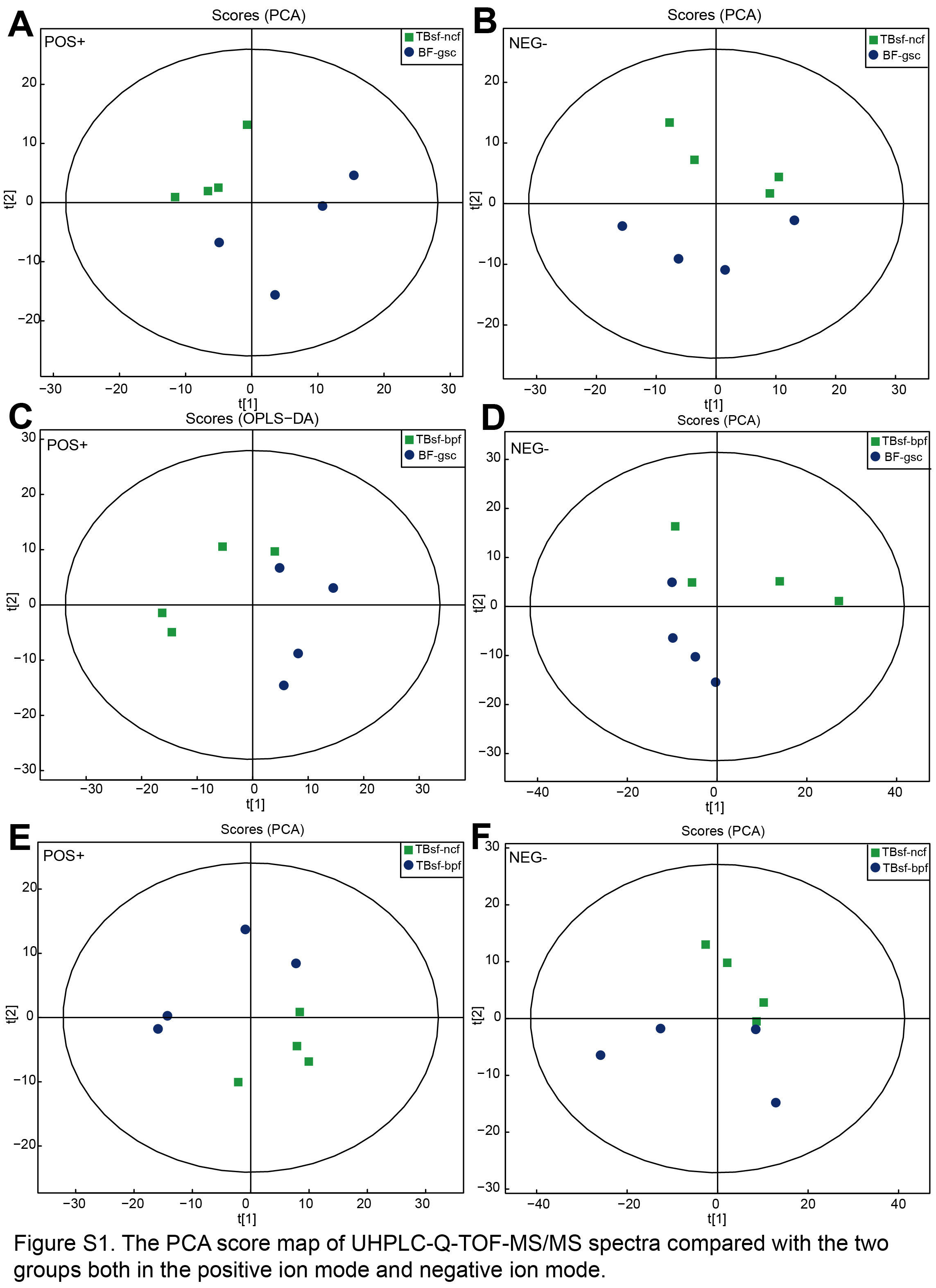

Supplement: Supplementary file 1 [file metabolites-12-00914-s001.zip › FigureS1.jpg]

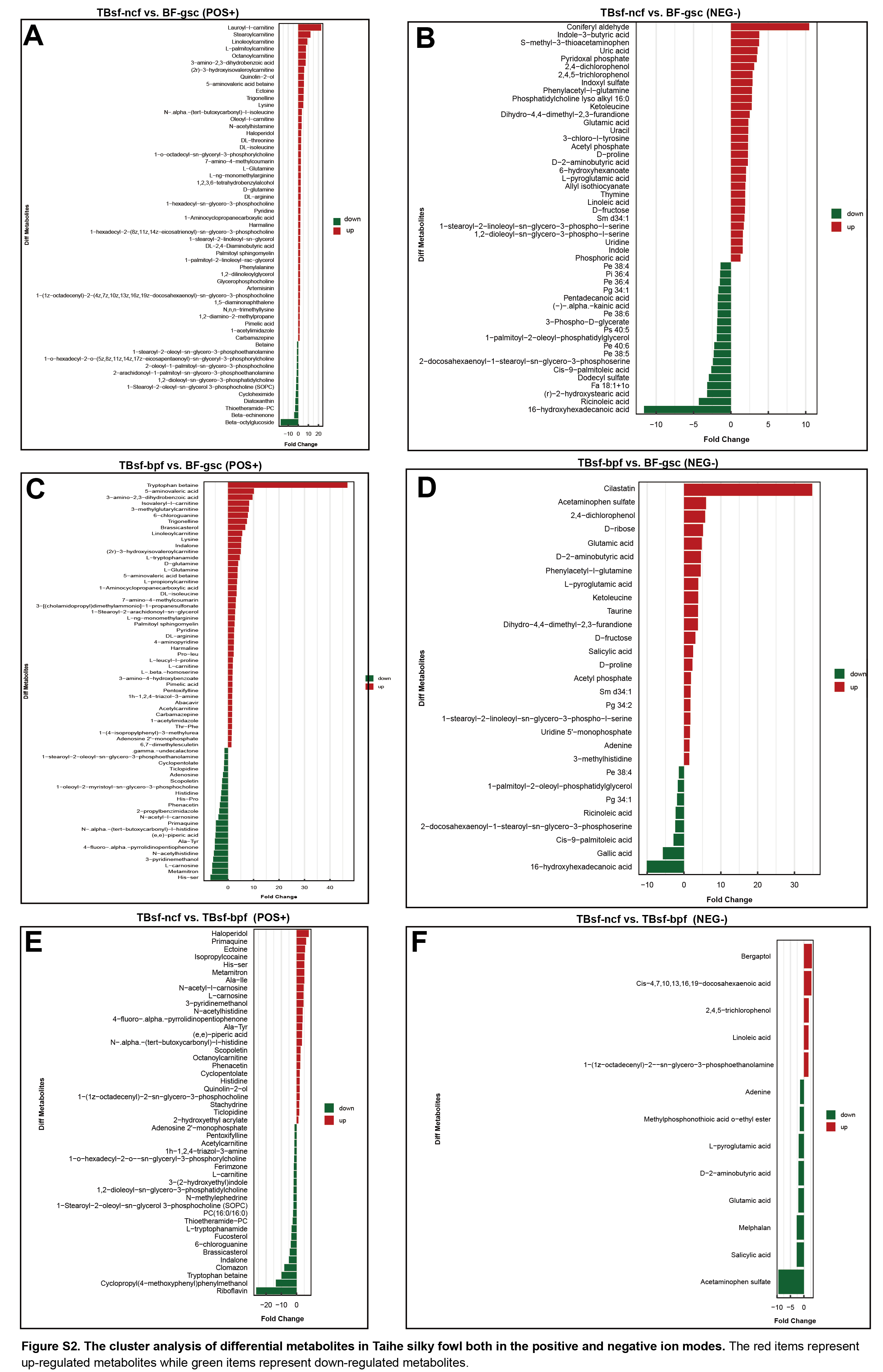

Supplement: Supplementary file 1 [file metabolites-12-00914-s001.zip › FigureS2.jpg]
